# Intimate partner violence and incidence of common mental disorder

**DOI:** 10.1590/S1518-8787.2017051006912

**Published:** 2017-03-29

**Authors:** Marcela Franklin Salvador de Mendonça, Ana Bernarda Ludermir

**Affiliations:** I Programa de Pós-Graduação Integrado em Saúde Coletiva. Universidade Federal de Pernambuco. Recife, PE, Brasil; II Departamento de Medicina Social. Centro de Ciências da Saúde. Universidade Federal de Pernambuco. Recife, PE, Brasil

**Keywords:** Battered Women, Mental Disorders, epidemiology, Intimate Partner Violence, Spouse Abuse

## Abstract

**OBJECTIVE:**

To investigate the association of intimate partner violence against women reported in the last 12 months and seven years with the incidence of common mental disorders.

**METHODS:**

A prospective cohort study with 390 women from 18 to 49 years, registered in the Family Health Program of the city of Recife, State of Pernambuco; from July 2013 to December 2014. The Self Reporting Questionnaire-20 (SRQ-20) assessed mental health. Intimate partner violence consists of concrete acts of psychological, physical or sexual violence that the partner inflicts on the woman. Poisson regression was used to estimate crude and adjusted relative risks (RR) of the association between common mental disorders and intimate partner violence.

**RESULTS:**

The incidence of common mental disorders was 44.6% among women who reported intimate partner violence in the last 12 months and 43.4% among those who reported in the past seven years. Mental disorders remained associated with psychological violence (RR = 3.0; 95%CI 1.9–4.7 and RR = 1.8; 95%CI 1.0–3.7 in the last 12 months, and seven years, respectively), even in the absence of physical or sexual violence. When psychological violence were related to physical or sexual violence, the risk of common mental disorders was even higher, both in the last 12 months (RR = 3.1; 95%CI 2.1–4.7) and in the last seven years (RR = 2.5; 95%CI 1.7–3.8).

**CONCLUSIONS:**

Intimate partner violence is associated with the incidence of common mental disorders in women. The treatment of the consequences of IPV and support for women in seeking protection for themselves for public services is essential.

## RESUMO

**OBJETIVO:**

Investigar a associação da violência por parceiros íntimos relatada contra as mulheres nos últimos 12 meses e últimos sete anos com a incidência dos transtornos mentais comuns.

**MÉTODOS:**

Estudo de coorte prospectivo com 390 mulheres de 18 a 49 anos, cadastradas no Programa Saúde da Família da cidade do Recife, PE, entre julho de 2013 e dezembro de 2014. A saúde mental foi avaliada pelo *Self Reporting Questionnaire*-20 (SRQ-20). A violência por parceiro íntimo foi definida por atos concretos de violência psicológica, física ou sexual infligidos à mulher pelo parceiro. A regressão de Poisson foi utilizada para estimar os riscos relativos (RR) brutos e ajustados da associação entre transtorno mental comum e violência por parceiro íntimo.

**RESULTADOS:**

A incidência dos transtornos mentais comuns foi de 44,6% entre as mulheres que relataram violência nos últimos 12 meses e de 43,4% nas que relataram violência nos últimos sete anos. Os transtornos mentais mantiveram-se associados à violência psicológica (RR = 3,0; IC95% 1,9–4,7 e RR = 1,8; IC95% 1,0–3,7 nos últimos 12 meses, e sete anos, respectivamente), mesmo na ausência de violência física ou sexual. Quando a violência psicológica esteve combinada com violência física ou sexual, o risco dos transtornos mentais comuns foi ainda mais elevado, tanto nos últimos 12 meses (RR = 3,1; IC95% 2,1–4,7) quanto nos últimos sete anos (RR = 2,5; IC95% 1,7–3,8).

**CONCLUSÕES:**

A violência por parceiro íntimo está associada à incidência de transtornos mentais comuns nas mulheres. É fundamental o tratamento das consequências da VPI e o apoio às mulheres na busca de proteção para si pelos serviços públicos.

## INTRODUCTION

Intimate partner violence (IPV) causes physical, sexual, or psychological harm, including physical assault, sexual coercion, psychological abuse, and controlling behaviors^a^. This occurs with more moderate situations in which diverse conflicts or episodes of frustration and anger occasionally erupt into aggression, and a more serious and chronic, progressive pattern^17^.

Acts of violence against women are not isolated events; this pattern of behavior violates the rights of women and girls, limits their participation in society and impairs their health and well-being^b^. The IPV complexity requires considering the structuring conditions of the Brazilian social fabric, validating the focus on the cultural context of the patriarchal system, which generates power asymmetry in human interactions, and intensifying conditions to worsening human rights violations^4^.

Dantas-Berger and Giffin^3^ consider that a social order of patriarchal tradition has long “consented” to a certain pattern of violence against women, assigning man the “active” role in the social and sexual relationships, while restricting the woman to passivity and reproduction. The male power game comes from these beliefs that men have rights and privileges more than women^4^.

IPV originates social and economic costs, harming the whole society^6^ ^,^ ^15^. Women may suffer isolation, inability to work, loss of wages and lack of participation in regular activities, and limit their ability to care for themselves and their children^a^.

The World Health Organization states that IPV is the most common type of violence against women, affecting 30% of women. It is a serious public health problem since it can lead to immediate injuries, infections and mental disorder^5^ ^,^ ^13^ ^,^ ^b^. The health sector has been slow to engage with violence against women.

Worldwide, mental health problems, emotional distress and suicidal behavior are common among women who have suffered IPV^7^ ^,^ ^c^. Common mental disorders (CMD) symptoms are insomnia, fatigue, irritability, forgetfulness, difficulty concentrating and somatic complaints, allied to depression and anxiety. CMDs cause a high social and economic cost due to lost days of work, as well as increasing health services demand^8^.

Using the Self Reporting Questionnaire-20 (SRQ-20)^10^, intimate partner violence, experienced by about 50% of Brazilian women, was associated with the CMD under analysis. The authors found a higher CMD among women who reported violence than among those who did not report violence (49% *versus* 19.6%, respectively). In addition, CMD increases with the severity of violence, with 30.6% for women who were victims of physical violence alone and 62.9% for those who suffered all forms of violence.

Women who have suffered IPV in the past may be more likely to have current psychological disorders than women who have never experienced IPV. The IPV effects on mental health can be immediate and acute, but also have long-term consequences or even become chronic^16^.

Estimating CMD in the population helps to understand its distribution in different groups in relation to its different characteristics, as well as the risk factors associated with its occurrence. The diagnosis provides relevant information to guide the policies of intervention in mental health, reducing or preventing worsening.

This study aimed to investigate the association of intimate partner violence reported against women in the last 12 months and the last seven years with the incidence of common mental disorders in women.

## METHODS

This is a prospective cohort study, part of a larger study we started in 2005, at the Distrito Sanitário II, in Recife, State of Pernambuco, and two stages are already completed. The first stage occurred from July 2005 to October 2006, when we contacted pregnant women during the prenatal visit. Of the 1,133 women eligible for the survey, we interviewed 1,120 (98.9%). The second stage occurred between three and six months postpartum. In the second stage, 1,057 women were interviewed, representing 94.3% of those who answered the first questionnaire.

This work is the third stage of the study. We invited all the women from the second stage to participate in this new phase, and they answered the same questions about violence that we did in the other stages, with reference to the last seven years and the last 12 months.

We used a structured questionnaire, in face-to-face interviews from July 2013 to December 2014 to data collection. The interviewers had higher education and experience in dealing with “violence against women”. There were weekly discussions with the interviewers in relation to the difficulties they found during the interviews. Most interviews occurred at the women’s home. The questions related to IPV were based on the questionnaire of the Multi-Country Study on Women’s Health and Domestic Violence, of the World Health Organization, validated in Brazil^18^.

The questions characterized physical assaults or the use of objects or weapons to cause injury as physical violence; as psychological violence, threatening behavior, humiliations and insults; and as sexual violence, sexual relations through physical force or threats and imposition of acts considered humiliating. Previous publication has details^10^. If the woman answered “yes” to at least one of the questions to each type of violence, we considered as a positive case.

The partner or ex-partner with whom the women lives or lived was her intimate partner regardless of formal union, including current boyfriends, as long as they had sexual relations.

The SRQ-20 instrument, developed by the World Health Organization, assessed mental health; it detects psychiatric problems in primary healthcare for developing countries^6^, with twenty yes-no questions, four physical symptoms and sixteen on psycho emotional disorders. The SRQ-20 was validated in Pernambuco with 62% sensitivity and 80% specificity^9^. In the data analysis, we assigned one point for each affirmative response and zero for each negative response. The SRQ-20 cut-off score was 7/8^6^ and the groups were non-suspected CMD (score ≤ 7) and suspected CMD (score ≥ 8).

The questions related to their demographic and socioeconomic characteristics: age (24-27; ≥ 28); race/ skin color (white; non-white); living with a partner (yes; no); years of education (0-4; ≥ 5); productive insertion (unemployed, others); monthly income (none; < 1 minimum wage^d^; ≥ 1 minimum wage). The data from the third Interview were typed in the EpiInfo program version 5.3.2, with double data entry and by different typists. Subsequently, the Validate software checked the typing errors and we cleaned and verified data consistency.

The Stata program version 10.1 for Windows performed statistical analysis. To test differences between proportions, we used the Chi-square test and considered those with p < 0.05 statistically significant. We used the Poisson regression to estimate the risk ratio (RR) and 95%CI of the association between CMD, socioeconomic and demographic characteristics of women, and types of IPV in the last 12 months and the last seven years. Potential confounding factors were chosen based on the analysis of the socioeconomic and demographic characteristics.

The Committee of Ethics and Research with Human Beings of the Universidade Federal de Pernambuco approved the study (Opinion 194.672), in accordance with Resolution 196/96, which regulates research with human beings. All participants signed a free and informed consent form, read at the beginning of the interview, when women received information about the location and coordination of the research, their voluntary and confidential nature, and the delicate and personal nature of some questions.

## RESULTS

We interviewed 60.9% (644) of the postpartum women who answered the second stage of the cohort. Six women had died. We lack to find 390 women due to change of address and 17 refused to remain in the survey. However, these 390 postpartum women did not show a statistically significant difference in relation to IPV and demographic and socioeconomic variables.

We excluded 254 women out of 644 in the current database who had CMD during pregnancy, resulting in a sample of 390.

In agreement with the socioeconomic and demographic characteristics, most women were over 28 years of age, non-white, living with a partner and having five or more years of education. Most women were also unemployed and received less than 1 minimum wage. The incidence of CMD in the last seven years was higher in women with monthly income less than 1 minimum wage (Table 1).

**Table 1:** Socioeconomic and demographic characteristics of women and their association with common mental disorders, risk ratio (RR), confidence intervals (95%CI) and p values.

Variable	n	%	Common mental disorder		RR (95%CI)	p

			n	%		
Age						
24-27	56	14.36	11	19.6	0.9 (0.5–1.6)	0.749
≥ 28	334	85.6	72	21.6	1.0	
Race/Skin color^a^						
White	70	18.0	14	20.0	1.0	
Non-white	318	81.9	68	21.4	1.1 (0.6–1.8)	0.799
Living with a partner						
Yes	311	79.7	64	20.6	0.8 (0.5–1.3)	0.496
No	79	20.3	19	24.0	1.0	
Years of education^b^						
0-4	55	14.2	15	27.8	1.0	
≥ 5	332	85.8	67	20.2	0.7 (0.4–1.2)	0.221
Productive insertion						
Unemployed	311	79.7	60	19.3	1.0	
Other	79	20.3	23	29.1	1.5 (0.9–2.3)	0.051
Monthly income						
None	35	9.0	5	14.3	0.9 (0.4–2.3)	0.853
Less than R$678.00	220	56.4	57	25.9	1.7 (1.1–2.6)	0.027
Equal or more than R$678.00	135	34.6	21	15.6	1.0	

^a^ Missing data from 2 participants.

^b^ Missing data from 3 participants.

In the 12 months prior to this study, 20.8% of women were victims of some type of violence. In relation to the last seven years, the victims of some type of violence totaled 26.2%. Among women who reported IPV, the most frequent violence was psychological violence. Currently, 21.3% of all women were affected by CMD (Table 2). The incidence of common mental disorders was 44.6% among women victims of violence in the last 12 months and 43.4% in those who reported violence in the last seven years (Table 3).

**Table 2:** Frequency of intimate partner violence in the last 12 months and last seven years and common mental disorders.

Variable	n*	%
IPV in the last 12 months		
No violence	309	79.2
Only psychological	37	9.5
Physical and sexual with or without psychological	44	11.3
IPV in the last seven years		
No violence	288	73.8
Only psychological	39	10.0
Physical and sexual with or without psychological	63	16.2
Common mental disorders		
During the pregnancy		
Yes	486	43.39
No	634	56.61
Currently		
Yes	83	21.3
No	307	78.7

IPV: intimate partner violence

* n during pregnancy = 1,120; n in the last 12 months and last seven years = 390.

**Table 3:** Incidence of common mental disorders in women victims of IPV in the last 12 months and the last seven years.

Variable	n	%	Crude RR	95%CI	Adjusted RR	95%CI*
IPV in the last 12 months						
No violence	46	55.4	1		1	
Only psychological	16	19.3	2.9	1.8–4.6	3.0	1.9–4.7
Physical and sexual with or without psychological	21	25.3	3.2	2.1–4.8	3.1	2.1–4.7
IPV in the last seven years						
No violence	47	56.6	1		1	
Only psychological	12	14.5	1.9	1.1–3.2	1.8	1.0–3.0
Physical and sexual with or without psychological	24	28.9	2.3	1.5–3.5	2.5	1.7–3.8

IPV: intimate partner violence

* Adjusted by the “productive insertion” and “monthly income” variables.

The risk ratio (RR) was higher in physical and sexual violence with or without psychological. The incidence of CMD showed a strong association with violence in the last 12 months (RR = 3.1; 95%CI 2.1–4.7) and in the last seven years (RR = 2.5; 95%CI 1.7–3.8), maintained even after adjusting RR for possible confounding factors (Table 3).

## DISCUSSION

This study estimated the incidence of common mental disorder in women victims of IPV, in which psychological violence was associated with CMD even when violence occurred without physical or sexual violence and after adjusting for other variables. The greatest association occurred in situations of physical and sexual violence with or without psychological violence in the last 12 months and in the last seven years, corroborating the findings of Ludermir et al.^10^, regarding the fact that CMD increases with the severity of violence. The small number of women victims of physical or sexual violence, similar to other studies^10^ ^,^ ^20^, made it impossible the analysis of these variables in isolation. To our knowledge, this is the first Brazilian cohort study estimating the incidence of CMD in women victims of IPV seven years after pregnancy.

According to our study, psychological violence was more common than physical and sexual violence and in women with low education and living with lower income, corroborating with other studies^6^ ^,^ ^10^ ^,^ ^11^ ^,^ ^15^. In fact, the frequency of IPV we found may reflect the characteristics of the territory of the study. Frequencies of violence also depend on the socioeconomic conditions of the population and on the sociocultural contexts in which the gender hierarchy is more or less legitimized, which contributes to the increase or decrease of violence reports^20^.

Exposure to violence is a common feature of women living in developing countries and is significantly associated with mental health problems^12^ ^,^ ^14^. This may happen because developing countries are conservative and patriarchal societies that reinforce gender inequality^2^. Women who are victims of IPV are more likely to have CMD symptoms^2^. In addition, a systematic review reported a moderate or strong association between IPV and depression, suggesting that women victims of IPV are about three times more likely to develop depression^19^.

Studies report that the more severe the aggression, the greater the impact on women’s mental health^12^ ^,^ ^15^. These studies are similar to our results, as we verified that the highest incidence of CMD occurred in women who reported more than one type of violence. This association may be a consequence of a greater frequency of violence against women, as well as of the more serious nature of violence, more common in situations with combined forms of violence^1^.

One of the limitations of this study is the information bias, regarding the underreporting of violence. Because it is a sensitive issue, stigma and shame can induce women to omit violence, leading to underreporting of the cases^10^. In addition, women’s difficulties and blockades to face the trauma and to remember this painful experience may interfere with their willingness to speak^20^. Thus, the results may be underestimated, leading to an underestimation of the strength of the association between violence and mental disorders.

The role of health professionals in identifying violence against women is still a matter of debate^20^. There is evidence that IPV is associated with the high frequency of demand for primary healthcare services^19^. Health professionals should be aware of women suffering from CMD as this may indicate that they are victims of IPV, as these two issues are strongly associated with the results in this study. In addition, IPV is an aggravation of compulsory notification of public health in public and private health services throughout the national territory, contemplated by Law 10.778/2003, which establishes compulsory notification of violence against women^e^.

Sometimes physical violence occurs with psychological violence, so health professionals who are treating victims of intimate partner physical violence should be sensitive to symptoms of potential mental health problems and refer them to appropriate mental health services^12^.

This study reinforces that IPV, often reported by women in Brazil, is associated with CMD. Public policies and strategies aimed at reducing gender-based violence can help prevent and reduce CMD symptoms among women who are victims of violence. It is also essential to address the IPV consequences and to support women in seeking protection in public services.

## Footnotes

a World Health Organization. Violence against women: intimate partner and sexual violence against women: updated November 2014 [cited 2016 Jun 22]. (Fact sheet N° 239). Available from: http://www.who.int/mediacentre/factsheets/fs239/en

b World Health Organization. Global and regional estimates of violence against women: prevalence and health effects of intimate partner violence and non-partner sexual violence. Geneva; 2013 [cited 2016 Dec 3]. Available from: http://apps.who.int/iris/

c World Health Organization. WHO multi-country study on women’s health and domestic violence against women: initial results on prevalence, health outcomes and women’s responses: report. Geneva; 2005 [cited 2016 Dec 3]. Available from: http://www.who.int/reproductivehealth/publications/violence/24159358X/en/

d It is equivalent to R$678.00 in the period of this study.

e Brasil. Lei nº 10.778, de 24 de novembro de 2003. Estabelece a notificação compulsória, no território nacional, do caso de violência contra a mulher que for atendida em serviços de saúde públicos ou privados. Brasília (DF); 2003 [cited 2014 Nov 3]. Available from: http://www.planalto.gov.br/ccivil_03/leis/2003/l10.778.htm
